# PAI1 inhibits the pathogenesis of primary focal hyperhidrosis by targeting CHRNA1

**DOI:** 10.1186/s13023-023-02808-0

**Published:** 2023-07-21

**Authors:** Jian-Feng Chen, Min Lin, Xu Li, Jian-Bo Lin

**Affiliations:** Department of Thoracic Surgery, the First Affiliated Hospital of Fujian Medical University, No. 20 Chazhong Road, Fuzhou, 350005 Fujian China

**Keywords:** PAI1, Hyperhidrosis, CHRNA1, AQP5, CACNA1C

## Abstract

**Background:**

Primary focal hyperhidrosis (PFH) may be attributed to the up-regulation of the cholinergic receptor nicotinic alpha 1 subunit (CHRNA1) in eccrine glands. Plasminogen activator inhibitor-1 (PAI1, encoded by *SERPINE1*) is reported to inhibit the expression of CHRNA1, while the role of PAI1 in hyperhidrosis is unknown.

**Methods:**

*Serpine1* KO mice, *Serpine1*-Tg mice, and wild type BALB/c mice were intraperitoneally injected with pilocarpine hydrochloride to induce PFH. Cisatracurium (CIS, antagonist of CHRNA1) or PAI-039 (small-molecule inhibitor of PAI1) was pre-administrated before the induction of hyperhidrosis. On the other hand, *Chrna1*-expressing AAV was constructed and administered to *Serpine1*-Tg mice with hydrochloride stimulation. Hydrochloride-related biomarkers, such as acetylcholine (ACH) in the serum, calcium voltage-gated channel subunit alpha1 C (CACNA1C), and aquaporin 5 (AQP5) in sweat glands of mice were assayed with ELISA, RT-PCR, and Western blot.

**Results:**

The administration of PAI-039 or *Pai1* knock-out increased *Chrna1* expression, sweat secretion, and hydrochloride-related biomarkers (ACH, CACNA1C, and AQP5) expression. On the other hand, CIS administration diminished the strengthened hyperhidrosis phenotype induced by *Pai1* knock-out with decreased sweat gland secretion.

**Conclusion:**

PAI1 inhibits CHRNA1-mediated hydrochloride-induced hyperhidrosis, with decreased sweat gland secretion and diminished ACH, AQP5, and CACNA1C expression. These results indicate the potential to utilize PAI1 to alleviate PFH.

## Background

Hyperhidrosis is a dermatosis pathologically characterized by excessive generalized or localized (focal) sweating. Focal hyperhidrosis (PFH) is the most common form, and exocrine glands are overactive due to sympathetic nervous system dysfunction not triggered by temperature or physical activity [1, 2]. The prevalence of PFH has been estimated to be 2-3% [3]. PFH patients are more prone to cutaneous infection, and clammy palms, soles, and axillae can seriously affect the life quality of PFH patients [4]. Thoracoscopic excision of the T2 and T3 sympathetic ganglia and radiofrequency ablation are suggested for both palmar and axillary hyperhidrosis-affected patients, while the long-term outcome is not satisfactory in considering compensatory hyperhidrosis at the back [5, 6]. Therefore, it is urgent to decipher more relevant mechanisms to find a curable treatment target.

Up-regulated choline acetyltransferase, acetylcholine (ACH) receptor subunits, and alpha-7 neuronal nicotinic receptor subunits are detected in the thoracic sympathetic ganglion, which indicates that cholinergic neuron dysregulation might contribute to the development of PFH [7]. Significantly up-regulated cholinergic receptor nicotinic alpha 1 subunit (CHRNA1) is observed in thoracic sympathetic ganglia, which can bind and gate the ACH neurotransmitter [8]. In our previous research, small-interfering RNA targeting *Chrna1* could attenuate the pathogenesis of pilocarpine-induced hyperhidrosis in mice [9]. On the other hand, we also found that cisatracurium pretreatment could block the function of CHRNA1 as an ion channel to prevent the pathogenesis of PFH in mice [10]. These studies demonstrate that CHRNA1 is a potential target to treat PFH.

Plasminogen activator inhibitor-1 (PAI1), encoded by *SERPINE1*, is a multi-functional protein, which can function as a principal inhibitor of plasminogen activation to inactivate both urokinase-type and tissue-type plasminogen activators [11]. On the other hand, PAI1 is a mechanistic contributor to several elements of the syndrome, including hepatic steatosis, and dyslipidemia, obesity, insulin resistance, and hypertension [12]. It is worth noting that PAI1 inhibitors (TM5484) could induce dose-dependent up-regulated *CHRNA1* expression in myoblastic cells to prevent sarcopenia progression [13]. Whether PAI1 could be the upstream molecule to modulate the expression of *CHRNA1* is still unknown. In this study, the potential role of PAI1 in targeting CHRNA1 to alleviate PFH is deciphered.

## Methods

### Hyperhidrosis mice model and treatment

*Serpine1* (PAI1) KO mice (JAX: 002507) and *Serpine1* (PAI1) transgenic mice (JAX: 007237) were ordered from Jackson Lab, and BALB/c mice (6–7 weeks old) were purchased from Peking Vital River Laboratory Animal Ltd. (Beijing, China). Six hours before hyperhidrosis induction, 1 mg/kg cisatracurium (CIS, Jiangsu Hengrui Pharmaceutical Company, Lianyungang, China) was injected intraperitoneally, 1 mg/kg PAI-039 (Sigma-Aldrich, St. Louis, MO) was administered orally, and 5 × 10^10^ µg *Chrna1*-expressing AAV (GenScript, Nanjing, China) were administrated as indicated. All experimental procedures were approved by the Animal Ethics Committee of Fujian Medical University (2020[328]).

2% iodine solution was smeared on the hind paws, and 5 mg/kg pilocarpine hydrochloride (Sigma-Aldrich) was immediately intraperitoneal injected to induce hyperhidrosis. When iodine solution was drying, 0.5 g/mL starch was utilized to cover the surface of the hind paws. Five min after perspiration stimulation, black spots on hind paws were imaged to assay hyperhidrosis with ImageJ software (National Institutes of Health). Two hours after hyperhidrosis induction, tissues and serum were harvested for further analysis.

### Western blotting

Lysed sweat gland tissues (50 µg), collected from three mice in each group after the examination of ACH secretion, were loaded on 12% SDS-PAGE and transferred to PVDF membranes. Nonspecific binding was blocked with 1% BSA for 1 h. The membranes were incubated with the primary antibodies specific for PAI1 (Abcam, cat#: ab28207), AQP5 (Abcam, cat#: ab78486), and CACNA1C (Abcam, cat#: ab84814) at a 1:1000 dilution at 4 °C overnight. The PVDF membranes were then incubated in peroxidase-conjugated secondary antibody (Invitrogen, Waltham, MA USA) at a 1:1000 dilution for 1 h and developed with an ECL system (GE Healthcare Life Sciences, Chalfont, UK). GAPDH was used as an internal control.

### qRT-PCR

Total RNAs were isolated from sweat gland tissues, collected from another three mice in each group after the examination of ACH secretion, with Trizol (ThermoFisher, Waltham, MA USA), which were further reversely transcribed into cDNA, and SYBR green kit (ThermoFisher) was utilized to detect the amplification with the following procedure: 95 °C for 10 min to denature, 40 cycles of 95 °C for 15 s to amplification, and 60 °C for 1 min to prolongate on a Mastercycler^®^ X50 Eppendorf thermocycler. *Gapdh* was used as an internal control. Primers were designed by Genscript Biotech Corporation (Nanjing, China) and validated by melt curve analysis, listed as follows:

*Chrna1*,

forward, 5’- TCATCATTCCCTGCCTGCTCTTCT-3’,

reverse, 5’-TCTCTGCAATGTACTTCACGCCCT-3’;

*Pai1*,

forward, 5’- TTCAGCCCTTGCTTGCCTC-3’,

reverse, 5’-ACACTTTTACTCCGAAGTCGGT-3’;

*Aqp5*,

forward, 5’-AGAAGGAGGTGTGTTCAGTTGC-3’,

reverse, 5’- GCCAGAGTAATGGCCGGAT-3’;

*Cacna1c*,

forward, 5’- ATGAAAACACGAGGATGTACGTT-3’,

reverse, 5’- ACTGACGGTAGAGATGGTTGC-3’;

*Gapdh*,

forward, 5’- AGGTCGGTGTGAACGGATTTG-3’,

reverse, 5’- TGTAGACCATGTAGTTGAGGTCA-3’.

### ELISA

Acetylcholine (ACH) level in the mice serum (six mice in each group) was assayed with the relevant ELISA kit (Abcam, ab287812, Cambridge, MA, USA) following manufacturer instructions. All standards and samples were measured with a Multiskan Mk3 Microplate Reader (Thermo Fisher) at a wavelength of 450 nm.

### Immunohistochemistry

Footpad skin tissues embedded in paraffin were sectioned (2 μm thickness) and processed with gradient alcohol and Antigen Retrieval Buffer (Abcam, ab93684). After blocking with 1% BSA, the sections were incubated with primary antibody against PAI1 (Abcam, Cat#: ab28207, 1:500), biotinylated secondary antibody, avidin: biotinylated enzyme complex, and 3,3′-diaminobenzidine substrates (Zhongshan Goldenbridge, Guangzhou, China). The sections were finally counterstained with hematoxylin.

### Statistical analysis

Data points represented the biological replicates. The differences between two groups were analyzed with Student’s t test. The differences between multiple groups were analyzed with one-way ANOVA followed by a post hoc test. All statistical analyses were performed using GraphPad Prism.

## Results

### Down-regulated PAI1 in sweat glands of hydrochloride-induced hyperhidrosis mice

**Fig. 1:**
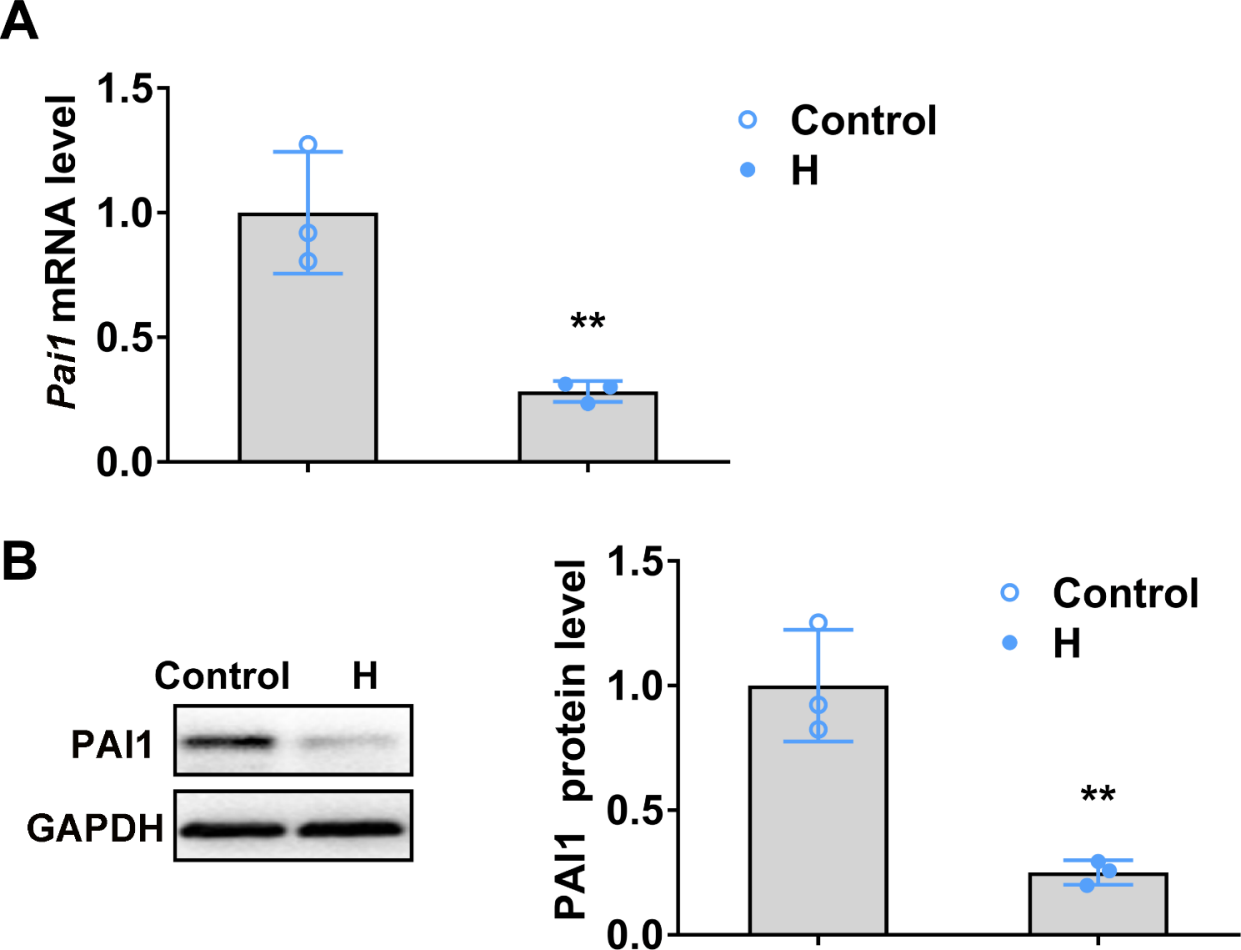
PAI1 was downregulated in sweat glands of hydrochloride-induced hyperhidrosis mice. The relative expression of PAI1 in both relative mRNA level **(A)** and relative protein level **(B)** in sweat glands of hydrochloride-induced hyperhidrosis mice were determined by RT-qPCR and Western blot, respectively. n = 3 mice. H, hyperhidrosis. The relative mRNA and protein expression level were normalized to control group. The significant difference was analyzed using two-tailed *t*-test. Error bar represents the Mean ± SD. **p < 0.01

In order to investigate the involvement of PAI1 in hyperhidrosis development, PAI1 expression in the sweat glands of hydrochloride-induced hyperhidrosis mice was detected with RT-PCR and Western blot. Diminished PAI1 expression in both mRNA (Fig. 1A) and protein (Fig. 1B) levels were detected.

### PAI1 inhibition increases CHRNA1 expression and sweat secretion in hydrochloride-induced hyperhidrosis mice

**Fig. 2:**
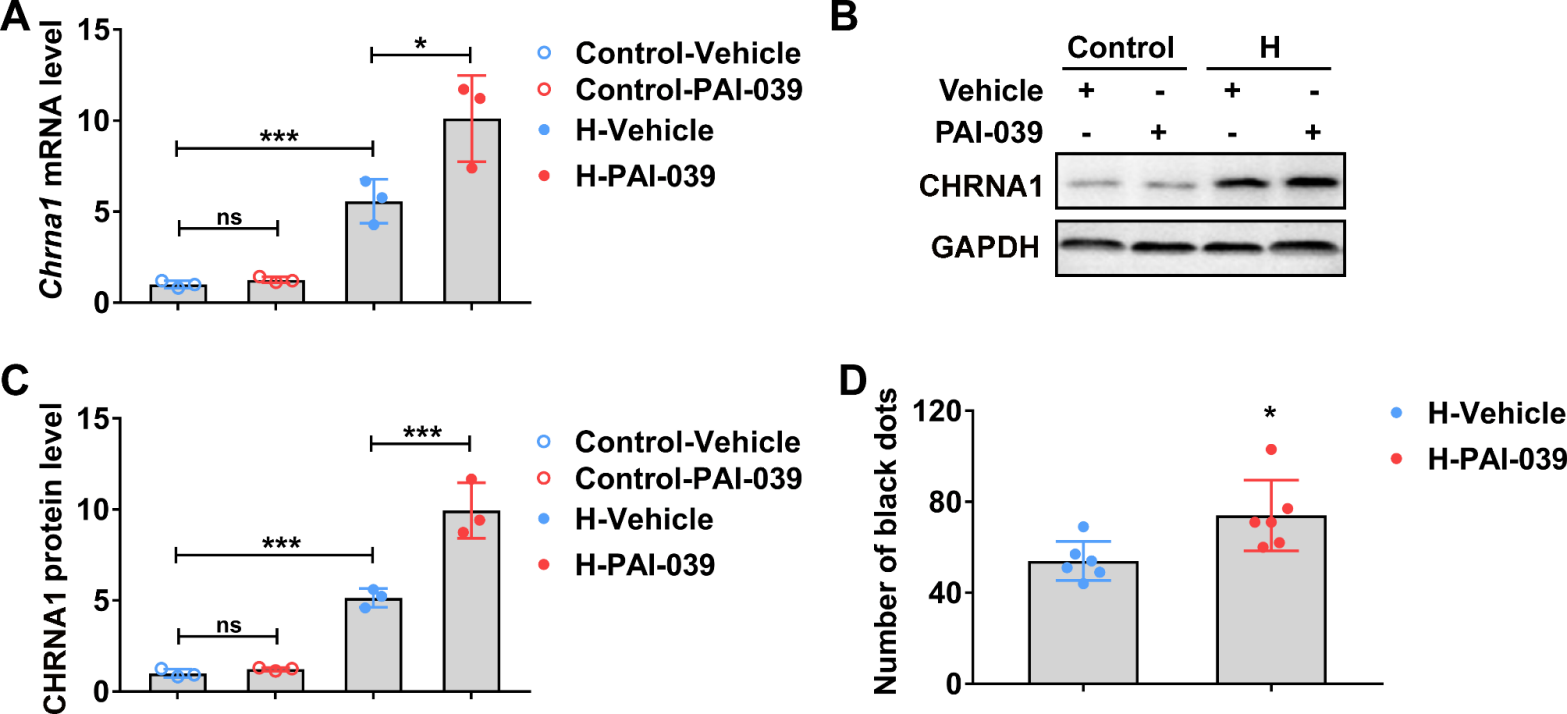
Inhibition of PAI1 increased the relative expression of CHRNA1 and sweat secretion in hydrochloride-induced hyperhidrosis mice. Mice were treated with 1 mg/kg PAI-039 or vehicle 6 h before the induction of hyperhidrosis. After hyperhidrosis induction, the relative CHRNA1 mRNA **(A)** and protein level **(B-C)** in sweat glands were detected by RT-qPCR and Western blot, respectively. n = 3. **(D)** The number of black dots was calculated with ImageJ. n = 6 mice. H, hyperhidrosis. The relative mRNA and protein expression level were normalized to control-vehicle group. The difference was analyzed by one-way ANOVA. Error bar represents Mean ± SD. *p < 0.05, ***p < 0.001. ns, not significant

PAI1 inhibitor (PAI-039) was utilized to decipher the role of PAI1 in hyperhidrosis. PAI-039 could up-regulate the relative expression of CHRNA1 in both mRNA (Fig. 2A) and protein levels (Fig. 2B C) in hydrochloride-induced hyperhidrosis mice, while PAI-039 did not alter the relevant CHRNA1 expression in normal mice without hydrochloride stimulation. On the other hand, PAI1 inhibition could functionally increase the extent of hyperhidrosis, as indicated by the increased number of black dots (Fig. 2D). These results indicated that PAI1 inhibition could promote hyperhidrosis phenotype and CHRNA1 expression.

### *PAI1* deficiency increases CHRNA1 expression in hydrochloride-induced hyperhidrosis mice

**Fig. 3:**
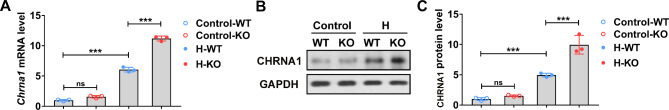
The deficiency of *Pai1* increased CHRNA1 expression in sweat glands of hydrochloride-induced hyperhidrosis mice. **(A-C)** The relative CHRNA1 mRNA **(A)** and protein level **(B-C)** in sweat glands of indicated groups were determined by RT-qPCR and Western blot, respectively. n = 3 mice. H, hyperhidrosis. The relative mRNA and protein expression level were normalized to control-WT group. The difference was analyzed using one-way ANOVA. Error bar represents Mean ± SD. ***p < 0.001. ns, not significant

A well-validated *Pai1* KO mice were used and confirmed by no detection of *Pai1* expression in foot pad skin (Fig. 3A) [14]. *Pai1* knock-out mice demonstrated up-regulated expression of CHRNA1 in both mRNA (Fig. 3A) and protein levels (Fig. 3B C) after hyperhidrosis induction. In contrast, *Pai1* knock-out mice without hyperhidrosis induction did not show any significant difference in the expression of CHRNA1 compared with normal control mice. These results indicated that *Pai1* knock-out could promote CHRNA1 expression in hydrochloride-induced hyperhidrosis mice.

### *Pai1* deficiency promotes CHRNA1-mediated hydrochloride-induced hyperhidrosis

**Fig. 4:**
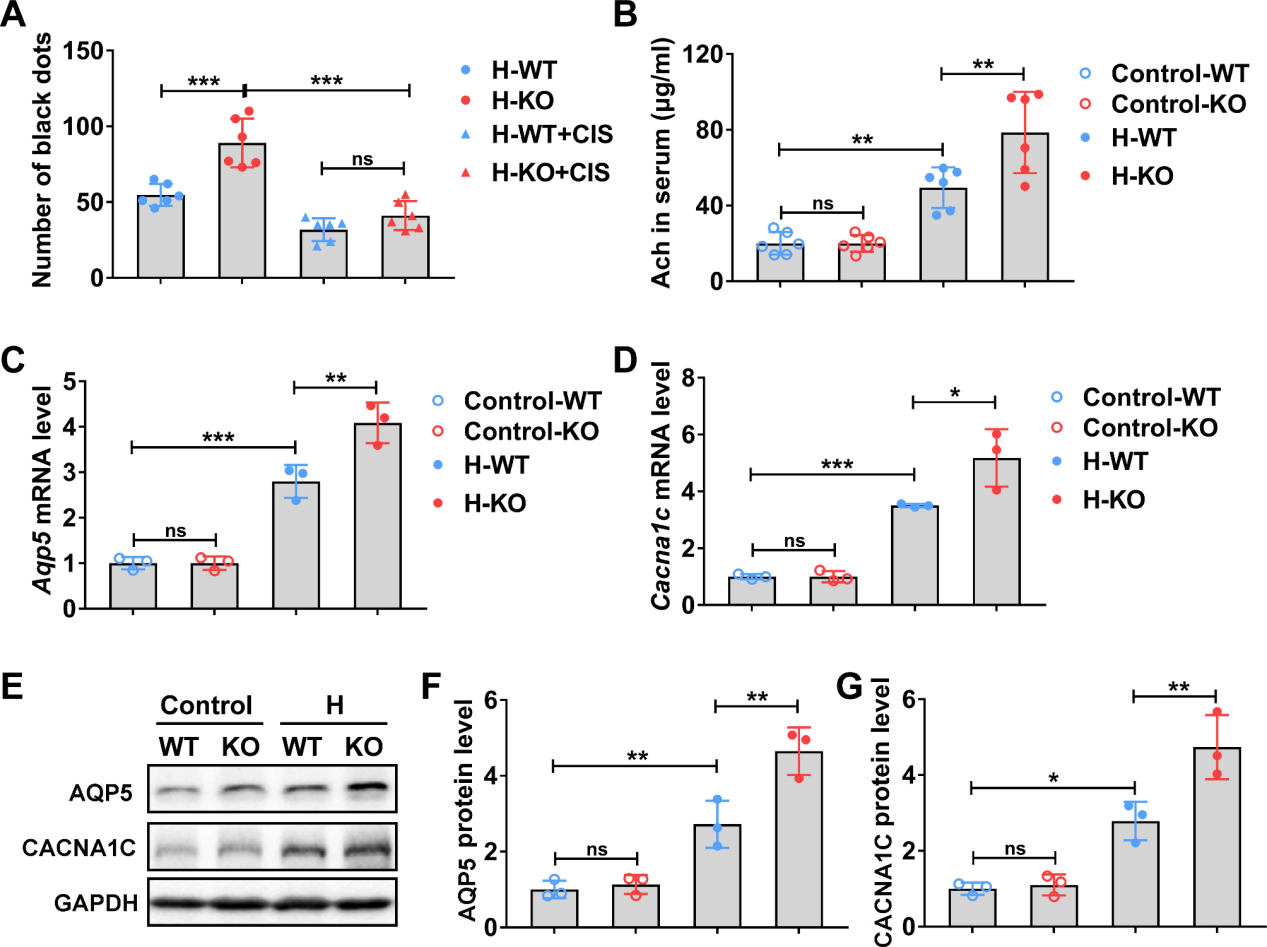
The deficiency of PAI1 promoted sweat secretion in hyperhidrosis mice. WT and *Pai1* KO mice were treated with vehicle (H-WT and H-KO groups) or cisatracurium (H-WT + CIS and H-KO + CIS groups) for 6 h, then were induced hyperhidrosis. After hyperhidrosis induction, the sweat secretion, serum acetylcholine level, and relative expression level of AQP5 and CACNA1 were analyzed. **(A)** After pilocarpine injection, black dots were calculated with ImageJ. n = 6 mice. **(B)** Acetylcholine concentration in serum was analyzed by ELISA. n = 6 mice. (C-D) The relative mRNA level of *Aqp5***(C)** and *Cacna1c***(D)** in sweat gland tissues were determined by RT-qPCR. n = 3 mice. **(E-G)** The relative protein level of AQP5 **(E and F)** and CACNA1C **(E and G)** in sweat gland tissues were determined by Western blot. n = 3 mice. H, hyperhidrosis. The relative mRNA and protein expression level were normalized to control-WT group. The difference was analyzed using one-way ANOVA. Error bar represents Mean ± SD. *p < 0.05, **p < 0.01, ***p < 0.001. ns, not significant

As an intermediate-acting, non-depolarizing neuromuscular blocking, CIS was utilized to down-regulation the relative expression of *Chrna1* as indicated by previous research [10]. *Pai1* knock-out mice demonstrated increased numbers of black dots, while such phenomenon could be prohibited by the administration of CIS (Fig. 4A), which indicated that PAI1 could negatively regulate CHRNA1-mediated hyperhidrosis. On the other hand, PFH-relevant molecules, such as ACH, AQP5, and CACNA1C, were detected. *Pai1* knock-out mice demonstrated up-regulated secretion of ACH in the serum (Fig. 4B), increased mRNA and protein levels of AQP5 (Fig. 4C and E, and 4 F), and CACNA1C (Fig. 4D and E, and 4G) after hydrochloride exposure. These results demonstrate that PAI1 could inhibit CACNA1C expression, which may contribute to the alleviation of hydrochloride-induced hyperhidrosis.

### Transgenic *Pai1* decreases CHRNA1-mediated hydrochloride-induced hyperhidrosis

**Fig. 5:**
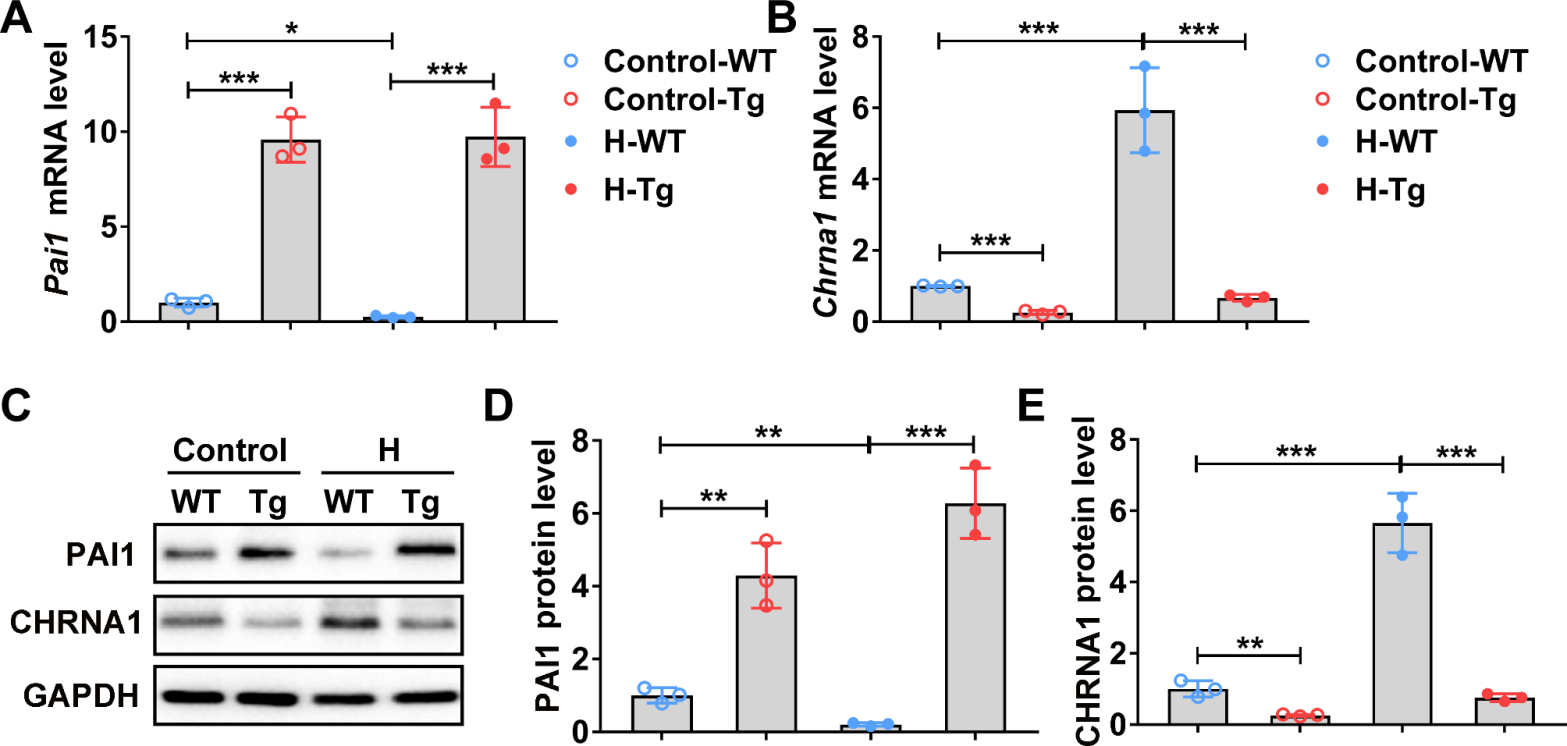
Transgenic *Pai1* decreased CHRNA1 expression in sweat glands of hydrochloride-induced hyperhidrosis mice **(A-B)** The relative mRNA level of *Pai1***(A)** and *Chrna1***(B)** in sweat glands of indicated groups were determined by RT-qPCR. **(C-E)** The relative protein level of PAI1 **(C and D)** and CHRNA1 **(C and E)** in sweat glands of indicated groups were determined by Western blot. n = 3 mice. H, hyperhidrosis. The relative mRNA and protein expression level were normalized to control-WT group. The difference was analyzed using one-way ANOVA. Error bar represents Mean ± SD. *p < 0.05, **p < 0.01, ***p < 0.001

To further reveal the role of PAI1, *Pai1*-Tg mice were used, in which *Pai1* expression was driven by a CMV promoter. Up-regulated *Pai1* expression was observed in *Pai1*-Tg mice (Fig. 5A and C, and 5D). As expected, *Pai1*-Tg mice showed diminished expression of CHRNA1 in both mRNA (Fig. 5B) and protein levels (Fig. 5C and E) after hydrochloride exposure in sweat glands.

**Fig. 6:**
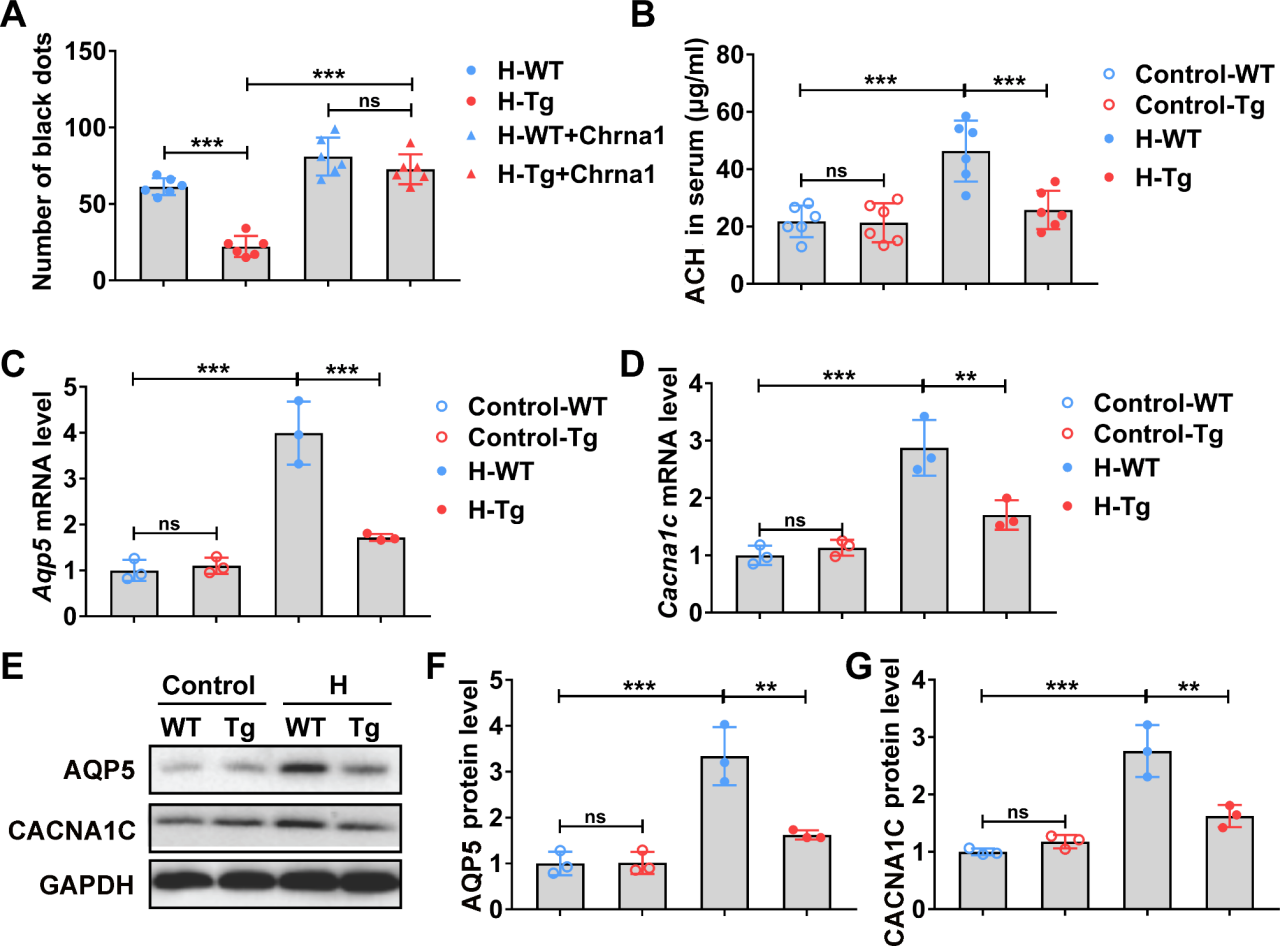
*Pai1* overexpression inhibited sweat secretion in hyperhidrosis mice. WT and *Pai1* transgenic (Tg) mice were treated with empty vector (H-WT and H-Tg groups) or *Chrna1*-expressing AAV (H-WT + Chrna1 and H-Tg + Chrna1 groups) for 72 h, then were induced hyperhidrosis. After hyperhidrosis induction, the sweat secretion, serum acetylcholine level, and relative expression level of *Aqp5* and *Cacna1* were analyzed. **(A)** Black dots were counted with ImageJ after pilocarpine injection. n = 6 mice. **(B)** Acetylcholine concentration in the serum was analyzed by ELISA. n = 6 mice. **(C-D)** The relative mRNA level of *Aqp5***(C)** and *Cacna1c***(D)** in sweat gland tissues were determined by RT-qPCR. n = 3 mice. **(E-G)** The relative AQP5 **(E and F)** and CACNA1C **(E and G)** protein level in sweat gland tissues were determined by Western blot. n = 3 mice. H, hyperhidrosis. The relative mRNA and protein expression level were normalized to control-WT group. The difference was analyzed using one-way ANOVA. Error bar represents Mean ± SD. **p < 0.01, ***p < 0.001. ns, not significant

*Pai1*-Tg mice showed a diminished number of black dots, which could be restored by the administration of *Chrna1*-expressing AAV (Fig. 6A). *Pai1*-Tg mice demonstrated down-regulated secretion of ACH in the serum (Fig. 6B) and down-regulated mRNA and protein levels of AQP5 (Fig. 6C and E, and 6 F) and CACNA1C (Fig. 6D and E, and 6G) in sweat glands of hydrochloride-induced hyperhidrosis when compared with normal mice exposure to hydrochloride. The above results further confirmed that transgenic *Pai1* could inhibit the development of CHRNA1-mediated hydrochloride-induced hyperhidrosis.

## Discussion

Down-regulated PAI1 is observed in sweat glands of hydrochloride-induced hyperhidrosis mice. PAI1 inhibition with PAI-039 or *Pai1* deficiency could induce up-regulated CHRNA1, ACH, AQP5, and CACNA1C expression and sweat secretion in hydrochloride-induced hyperhidrosis mice. On the other hand, transgenic *Pai1* could inhibit the development of hyperhidrosis with decreased CHRNA1 expression, and the alleviation of hyperhidrosis phenotype could be restored by the administration of *Chrna1*-expressing AAV. Our results indicate that PAI1 could prohibit CHRNA1-mediated hydrochloride-induced hyperhidrosis.

Up-regulated CHRNA1 could be considered as a characteristic biomarker in PFH patients sweat glands [9]. CHRNA1 homeostasis is vital for postsynaptic signal regulation, and altered CHRN endocytosis mediated by carbonic anhydrase 3 might contribute to the pathogenesis of myasthenia gravis [15]. Postsynaptic CHRNA1 ablation could preserve muscle innervation in type III neuregulin 1 mutant mice [16]. On the other hand, pharmacological inhibition of PAI1 with tiplaxtinin could block lysosomal degradation to up-regulate surface PDL1 expression in melanoma to synergize PDL1 immune checkpoint blockade [17], which indicates that PAI1-mediated endocytosis is a novel mechanism to regulate the homeostasis of signal molecules. Although the detailed or precise mechanism of PAI1-mediated CHRNA1 inhibition is not deciphered in this study, our investigation further indicates that PAI1-mediated endocytosis may affect CHRNA1 homeostasis in hyperhidrosis. Further understanding of CHRNA1 homeostasis-mediated by PAI1 will shed light on the pathogenesis of hyperhidrosis. In addition, PAI1 may also bind to low-density lipoprotein receptor-related protein 1 to trigger JAK/STAT1 signaling [18]. Whether such a mechanism is also involved in hyperhidrosis needs further analysis.

PAI1 regulation could alter the relative expression of CACNA1C and AQP5, which are vital components involved in the process of sweat secretion and pathological hyperhidrosis. AQP5, an exocrine-type water-channel protein, may mediate the excretion of water from sweat gland [19, 20]. In *Aqp5* knock-out mice, decreased number of active sweat glands is observed upon pilocarpine stimulation [21]. CACNA1C, as a subunit of voltage-dependent calcium channel, may mediate calcium ions influx into the cell upon membrane polarization [22, 23]. In addition to the fact that serum ACH level can be regulated by PAI1, it is indicated that PAI1 has an inhibitory effect on neurosecretion.

The precise mechanism related to PAI1 and CHRNA1 is not deciphered in this study. PAI1 may contribute to the development of thrombosis, fibrosis, obesity, and insulin resistance [24, 25]. PAI1 not only functions as a plasminogen activator inhibitor, but also interacts with vitronectin to promote the development of multiple cancers, thrombotic and vascular disorders [26, 27]. In addition, the function of PAI1 in the intravascular space differ from its role in the brain parenchyma under both pathological and physiological conditions [28]. Further research on the utilization of PAI1 in hyperhidrosis will depend on a detailed understanding of PAI1 interaction with vitronectin or the administration sites. For the indicated neurosecretion mechanism and potential plasminogen activator inhibitor, the clinic safety or route of administration may be a major consideration in the future.

There are two limitations should be noted. First, to obtain a more convincing conclusion, more samples in each group should be included. Meanwhile, it may introduce bias using Student’s t test for comparisons in a small number of samples. Second, studies on detailed mechanisms are warranted to understand the function of PAI1 in PFH.

## Conclusion

PAI1 inhibition and *Pai1* deficiency will promote CHRNA1 expression and pathogenesis of PFH. On the other hand, *Pai1* transgenic could inhibit the expression of CHRNA1 and the pathogenesis of PFH. Such effect could be restored by the administration of *Chrna1*-expressing AAV. Our findings demonstrate that PAI1 could target CHRNA1-mediated hydrochloride-induced hyperhidrosis in mice.

## Data Availability

The datasets used and/or analyzed during the current study are available from https://pan.baidu.com/s/1t5hRIaU7AoX3cv_P3BXbBA?pwd=3v9z.

## Abbreviations

PFH: Primary focal hyperhidrosis
CHRNA1: Cholinergic receptor nicotinic alpha 1 subunit
PAI1: Plasminogen activator inhibitor-1
ACH: Acetylcholine
CACNA1C: Calcium voltage-gated channel subunit alpha1 C
AQP5: Aquaporin 5
